# Supplementary figures and images for: Succinate dehydrogenase activity supports de novo purine synthesis
Source: bioRxiv. 2025 Mar 1:2025.02.26.640389. Preprint. [Version 1] doi: 10.1101/2025.02.26.640389 (PMC11888382; doi:10.1101/2025.02.26.640389)

# Supplementary Fig. 1

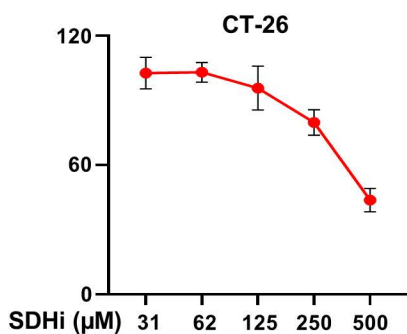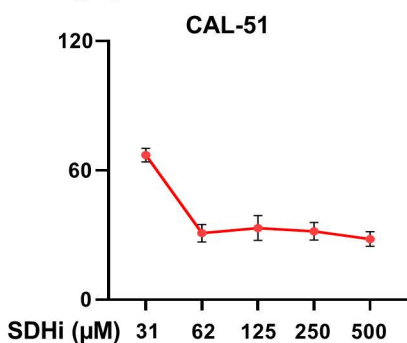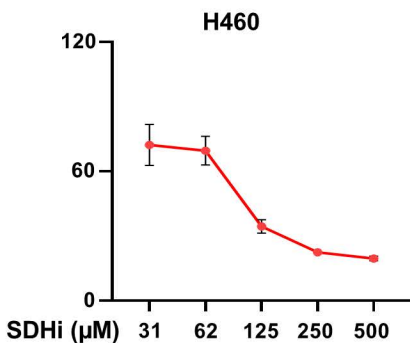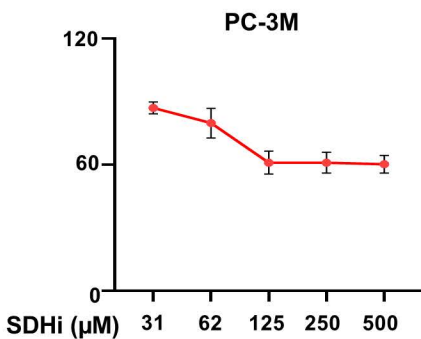

**A**

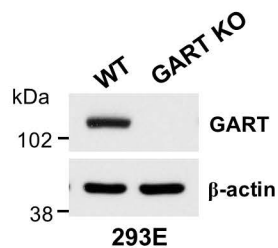

**B**

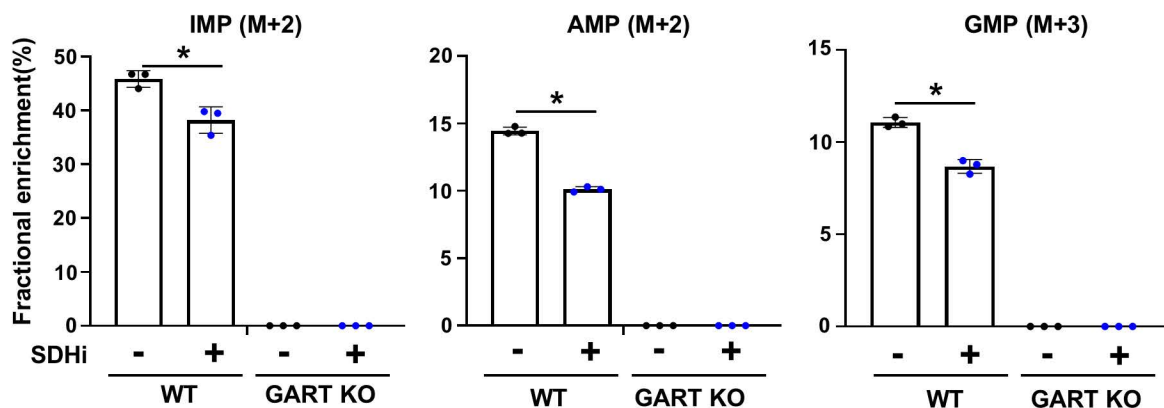

**C**

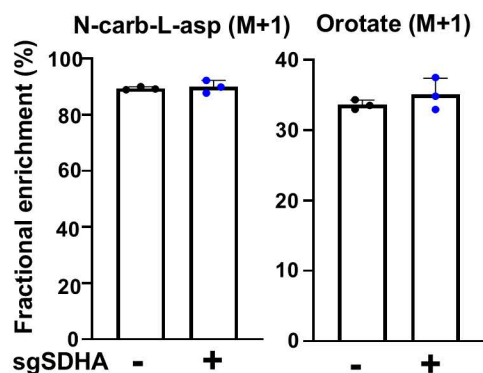

**D**

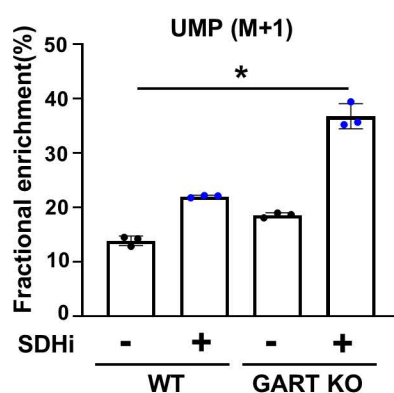

**E**

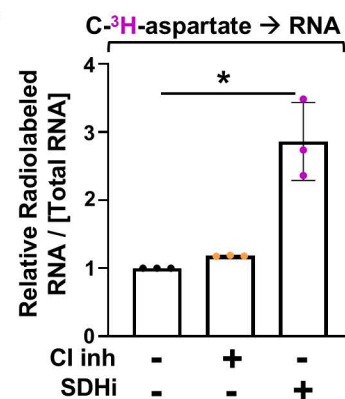

**F**

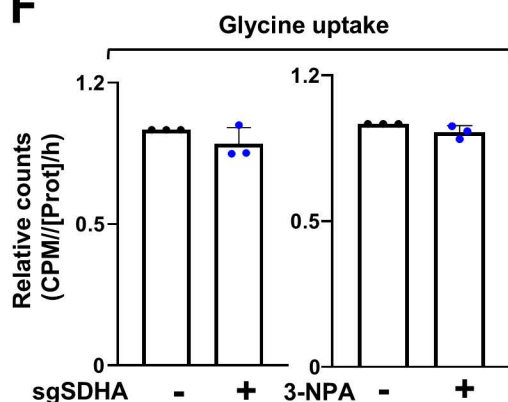

**G**

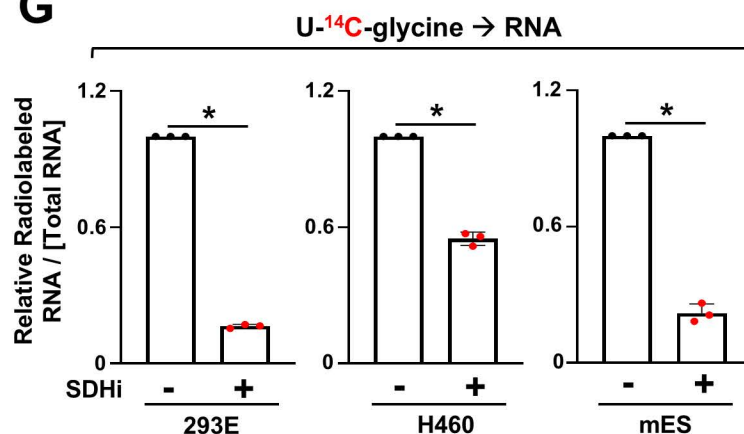

**H**

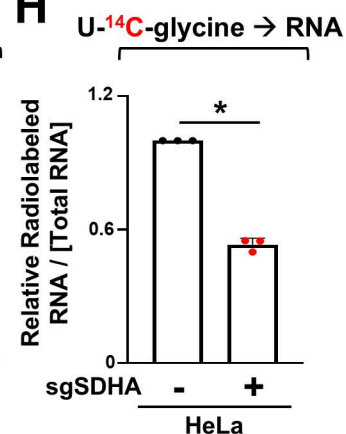

**I**

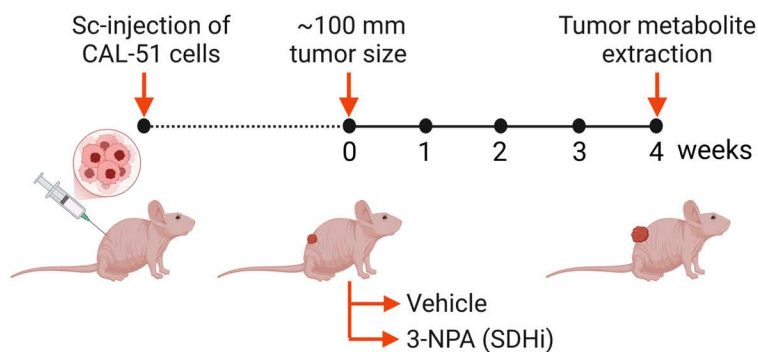

**J**

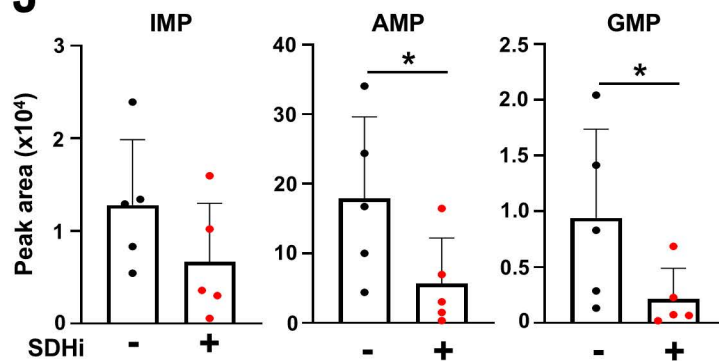

# Supplementary Fig. 3

**A**

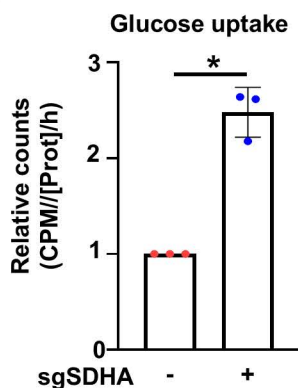

**B**

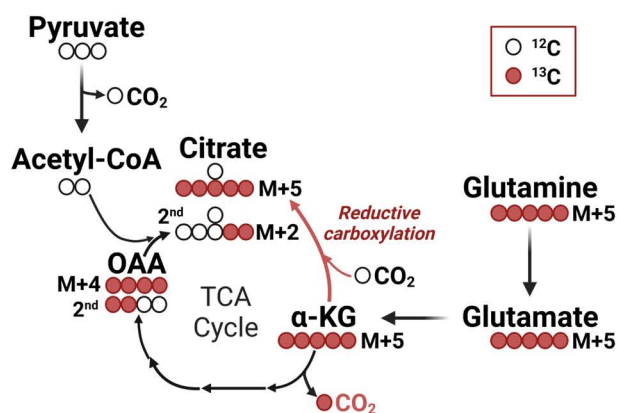

**C**

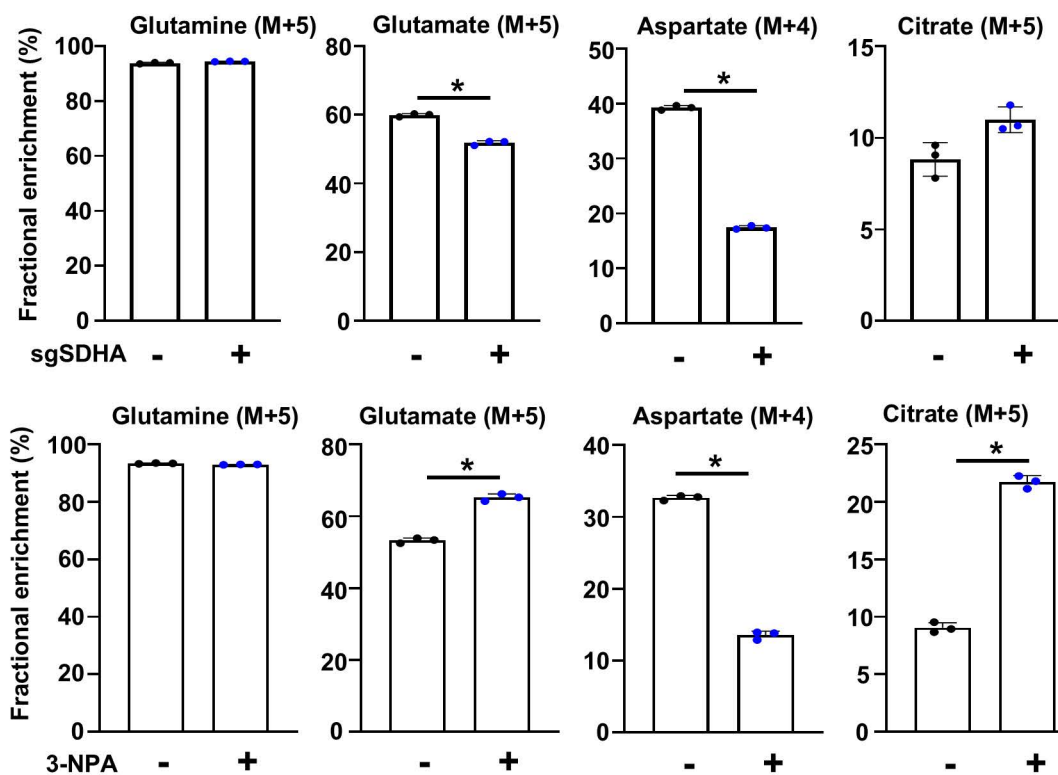

**D**

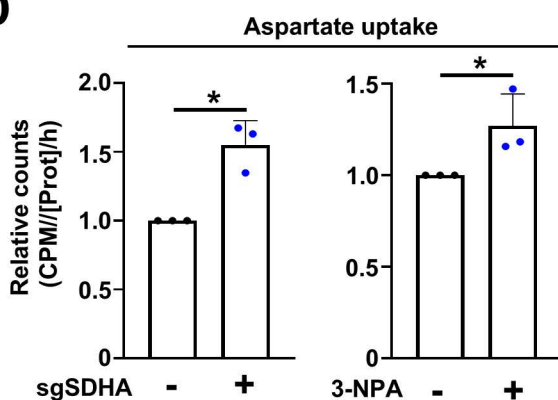

**A**

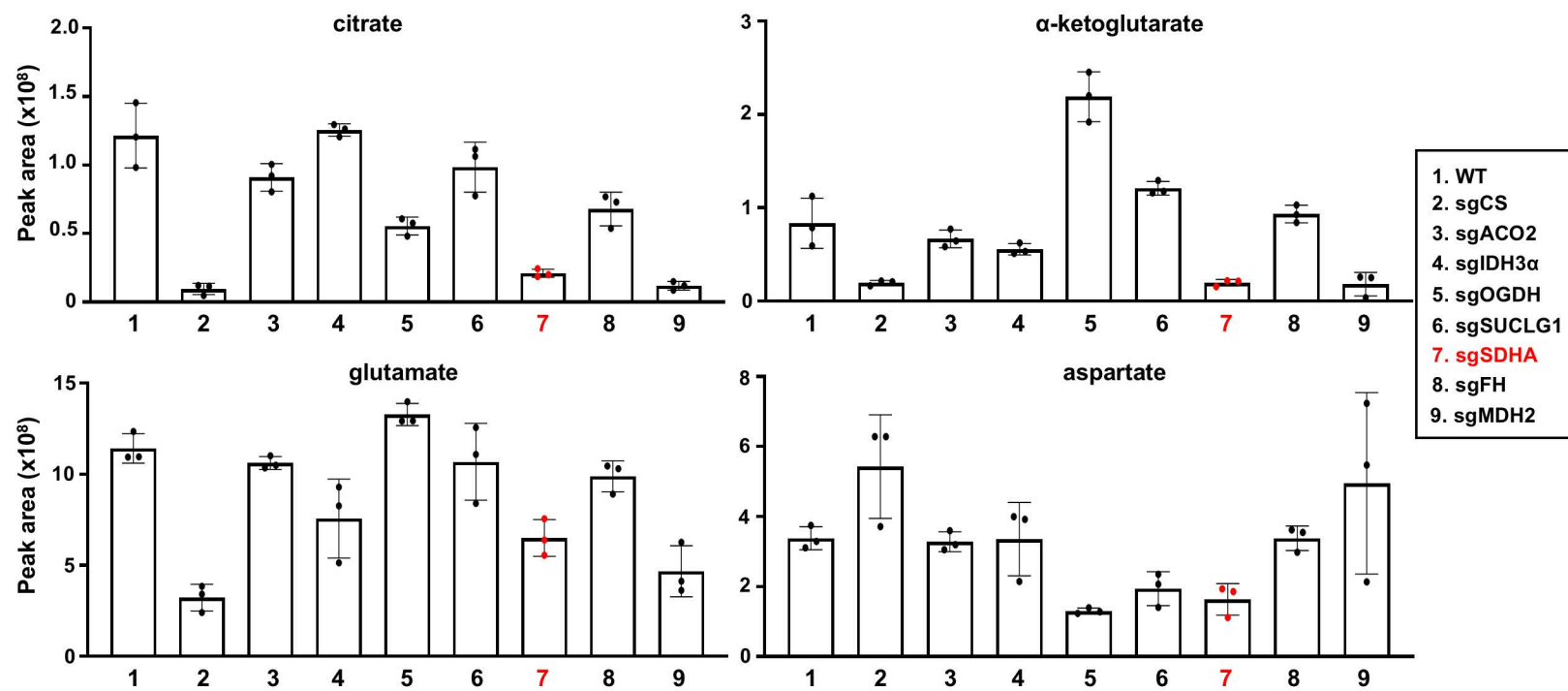

**B**

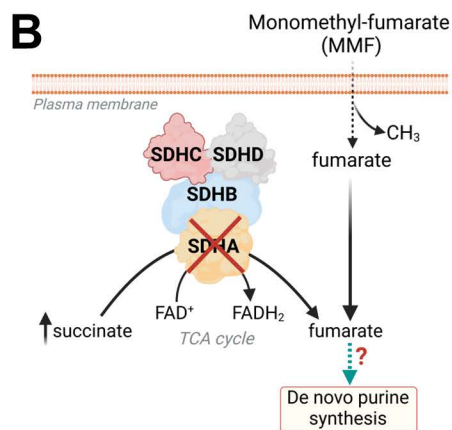

**C**

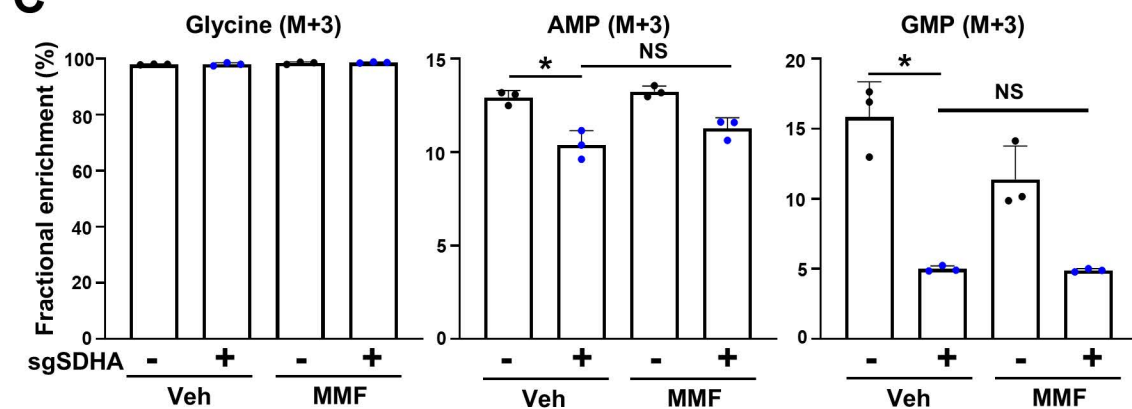

**D**

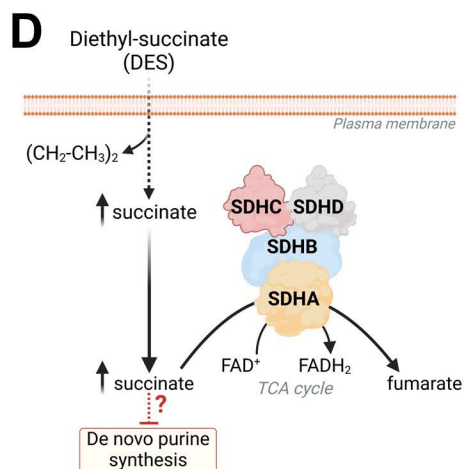

**E**

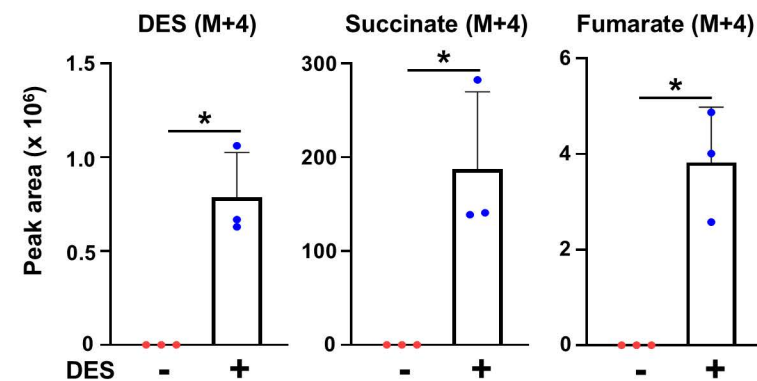

**F**

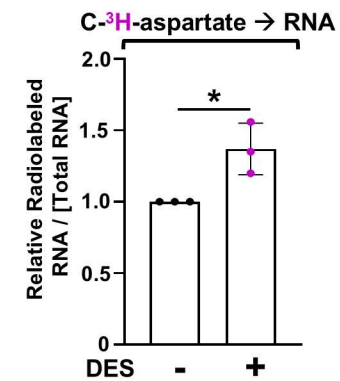

# Supplementary Fig. 5

## A

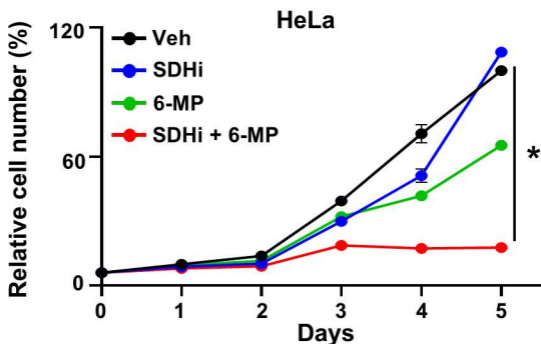

## B

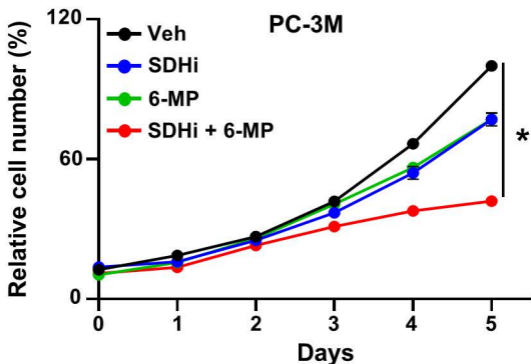

Supplement: 1 — Supplementary Fig. 1. Dose-dependent SDH inhibition by 3-NPA reduces proliferation in multiple cancer cell lines. Shown are dose–response curves for 3-NPA (SDHi) treatment in CT-26, CAL-51, H460, and PC-3M cells, illustrating decreased cell proliferation with increasing inhibitor concentrations. Data represent mean ± s.d. from n = 3 independent biological replicates. Supplementary Fig. 2. SDH inhibition leads to purine synthesis suppression. (A) Immunoblots of HEK 293E WT cells or knockout for the purine enzyme GART (sgGART). (B) Fractional enrichment (%) of the indicated metabolites from wildtype or GART KO HEK-293E cells and labeled with [15N-13C2]–glycine (400 μM) for 4 h and treated either with vehicle (DMSO) or 3-NPA. (C) Fractional enrichment (%) of the indicated pyrimidine intermediates from wildtype or SDHA KO (sgSDHA) HeLa cells labeled with [15N-(amide)]-glutamine (4 mM) for 2 hours. (D) Fractional enrichment (%) of UMP (M+1) from wildtype or GART KO HEK-293E cells treated with vehicle (DMSO) or 3-NPA (SDHi, 1 mM) and labeled with [15N-(amide)]-glutamine (4 mM) for 2 hours. (E) The relative levels of incorporation of [3H] from [C-2,3-3H]-aspartate into RNA are shown. Labeling in HeLa cells was performed for 6 hours treated with either vehicle (DMSO), rotenone (CI inh, 1μM), or 3-NPA (SDHi, 1 mM). Radioactivity measured reflects de novo pyrimidine synthesis activity. (F) Uptake of glycine in wildtype or SDHA KO HeLa cells or wildtype HeLa cells treated with vehicle (DMSO) or 3-NPA for 8 hours and labeled with [U-14C]-glycine for 5 min. (G) The relative levels of incorporation of [14C] from [U-14C]-glycine into RNA are shown. Labeling was performed in HEK 293E, H460 (lung cancer) and mouse embryonic stem (mES) cells, treated either with DMSO or 3-NPA for 9 hours. Radioactivity measured reflects de novo purine synthesis activity. (H) The relative levels of incorporation of [14C] from [U-14C]-glycine into RNA are shown. Labeling in wildtype and SDHA KO HeLa ce [file NIHPP2025.02.26.640389V1-supplement-1.pdf]
